# Immunotherapy Advances for Epithelial Ovarian Cancer

**DOI:** 10.3390/cancers12123733

**Published:** 2020-12-11

**Authors:** Erin G. Hartnett, Julia Knight, Mackenzy Radolec, Ronald J. Buckanovich, Robert P. Edwards, Anda M. Vlad

**Affiliations:** 1 Department of Obstetrics and Gynecology and Reproductive Sciences, Magee-Womens Research Institute and Foundation and Magee-Womens Hospital of UPMC, School of Medicine, University of Pittsburgh, Pittsburgh, PA 15213, USA; EHartnett@Wihri.org (E.G.H.); radolecm@upmc.edu (M.R.); buckanovichrj@mwri.magee.edu (R.J.B.); edwarp@upmc.edu (R.P.E.); 2 School of Medicine, University of Pittsburgh, Pittsburgh, PA 15213, USA; Knight.Julia@medstudent.pitt.edu

**Keywords:** epithelial ovarian cancer, immune therapy, cancer vaccines, immune checkpoint inhibitors, CAR T cells, oncolytic viruses

## Abstract

**Simple Summary:**

The overall five-year survival rate in epithelial ovarian cancer is 44% and has only marginally improved in the past two decades. Despite an initial response to standard treatment consisting of chemotherapy and surgical removal of tumor, the lesions invariably recur, and patients ultimately die of chemotherapy resistant disease. New treatment modalities are needed in order to improve the prognosis of women diagnosed with ovarian cancer. One such modality is immunotherapy, which aims to boost the capacity of the patient’s immune system to recognize and attack the tumor cells. We performed a retrospective study to identify some of the most promising immune therapies for epithelial ovarian cancer. Special emphasis was given to immuno-oncology clinical trials.

**Abstract:**

New treatment modalities are needed in order to improve the prognosis of women diagnosed with epithelial ovarian cancer (EOC), the most aggressive gynecologic cancer type. Most ovarian tumors are infiltrated by immune effector cells, providing the rationale for targeted approaches that boost the existing or trigger new anti-tumor immune mechanisms. The field of immuno-oncology has experienced remarkable progress in recent years, although the results seen with single agent immunotherapies in several categories of solid tumors have yet to extend to ovarian cancer. The challenge remains to determine what treatment combinations are most suitable for this disease and which patients are likely to benefit and to identify how immunotherapy should be incorporated into EOC standard of care. We review here some of the most promising immune therapies for EOC and focus on those currently tested in clinical trials.

## 1. Introduction

Epithelial ovarian cancer (EOC), the most common form of ovarian cancer, is the deadliest gynecologic cancer and the fifth leading cause of cancer death in women, with estimated 22,530 new cases and 13,980 deaths in 2019 [1]. According to morphological and clinical characteristics, EOC includes several subtypes (serous, endometrioid, clear cell, mucinous, transitional cell carcinomas) that represent different diseases, with different pathogenic mechanisms. High grade serous ovarian cancer (HGSOC), believed to originate primarily from the fallopian tubes [2,3], is the most prevalent histotype, accounting for 50–60% of all EOC [4]. The overall five-year survival rate in EOC is 44% and has only marginally improved since the introduction of platinum-based chemotherapy [1,5]. Despite an initial response to standard treatment with aggressive surgical debulking and platinum/taxane drug combination, the tumors invariably recur, and patients ultimately die of platinum-resistant disease. Over the past 20 years, there has been minimal progress regarding the overall survival of patients with EOC. Therefore, new treatment modalities are needed in order to improve the prognosis of women diagnosed with this disease.

Despite the widely accepted view that the diagnostic entity of EOC refers to a mixture of different diseases, with varying etiology and disease pathogenesis, most patients continue to receive the same, standard treatment regimen. With the advent of complex profiling technologies, optimized therapies may ultimately follow the molecular stratification of tumors for “personalized” approaches. While a wide variety of targeted therapies are under investigation, thus far, only two types of molecularly targeted agents have been licensed for therapy of EOC in the past decade [6]; bevacizumab, a monoclonal antibody targeting VEGF, and more recently olaparib, rucaparib and niraparib, all oral inhibitors of poly(ADP-ribose) polymerase (PARP) that have received FDA approval for varying indications in the treatment of EOC [7]. As we better understand the immune profile of the tumor microenvironment, one promising approach is the development of combinations of these established therapeutic agents with immunotherapy agents [8]. The field of immuno-oncology has experienced remarkable progress in recent years, primarily due to therapeutic advancements seen in melanoma, lung, urothelial and head and neck cancers. Despite limited clinical benefit achieved in EOC early trials, there is reason to believe that the promising results seen with immunotherapies in other cancers will also extend to EOC [9].

**Figure 1:**
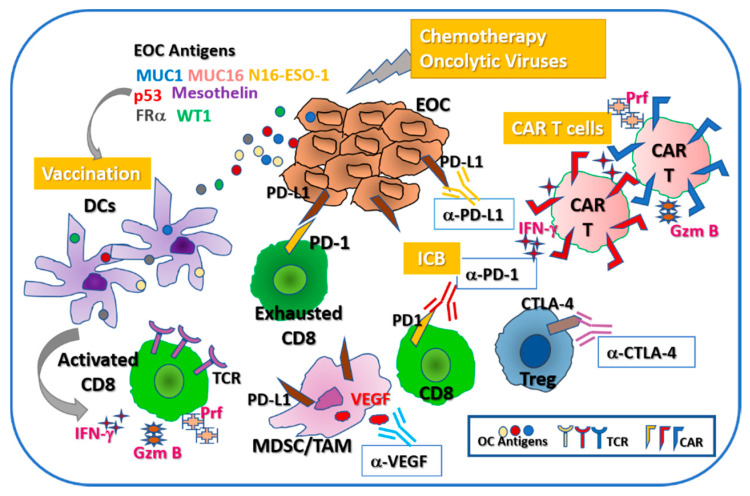
Diagram summarizing the main immune mechanisms targeted by immune therapy in epithelial OC. Induction of tumor cytolytic effects (via chemotherapy of oncolytic viruses) leads to the release of tumor antigens that are taken up by antigen presenting cells, primarily dendritic cells. Following intracellular processing, peptide epitopes are presented to MHC class I and class II restricted T cells. Tumor antigen primed, activated CD8 T cells express cytotoxic markers IFN-g, Gzm B and Prf, which are essential for tumor cell killing. Tumor antigen-loaded DC vaccines (using antigens such as MUC1, MUC16, NY-ESO-1, p53, mesothelin, FRa, WT1) have been developed for EOC treatment. To become effective against tumor cells, the tumor antigen specific, activated effector (especially CD8) T cells need to overcome tumor-induced immune suppressive interactions, including those occurring via immune checkpoint molecules like PD-1 (on T cells) and PD-L1 (on tumor cells). Immune checkpoint blockade (ICB) using blocking antibodies (a-PD-1, a-PD-L1, a-CTLA-4) can release the “break” on and revert “exhaustion” of TILs. Cells such as MDSC and Tregs carry out additional immune suppressive effects that can be targeted via VEGF blockade and CTLA-4 blockade, respectively. Newly engineered CAR T cells recognize tumor antigens in a non-MHC restricted manner and are cytotoxic against tumor cells. Abbreviations: CAR—chimeric antigen receptor; EOC—epithelial ovarian cancer; FRα—folate receptor alpha; Gzm B—granzyme B; MDSC—myeloid derived suppressor cells; MHC- major histocompatibility complex; MUC1—mucin 1; Prf—perforin; TAM—Tumor associated macrophages; TCR—T cell receptor; TILs—tumor infiltrating lymphocytes; Treg—regulatory T cells; VEGF—vascular endothelial growth factor; WT1—Wilms tumor 1.

In general, the overall goal of immune therapy alone or in combination with other targeting agents is to: (i) trigger robust immune effector mechanisms (mainly cytotoxic CD8 T cells) that can exert their cytolytic function against tumor cells via several mechanism (including IFN-γ, perforin, granzyme B); (ii) overcome immune suppression from tumor resident cells such as FOXP3+ immune regulatory T cells (Tregs) and immature myeloid derived suppressor cells (MDSC); (iii) avert immune checkpoint mechanisms responsible for immune exhaustion (via molecules such as PD-1/PD-L1, CTLA-4 etc.) (Figure 1). We review here some of the most promising immune therapies and focus on those currently tested in clinical trials.

## 2. EOC Immune Landscape

Numerous studies have demonstrated that the immune system plays an important role in the development and progression of EOC. Early studies reported that CD3+ tumor-infiltrating lymphocytes (TILs) were present in 55% of EOC and that the presence of TILs was correlated with improved clinical prognosis [10]. BRCA1-deficient tumors exhibit particularly high numbers of TILs, partly explaining the improved outcome in these patients [11,12]. Further investigation into the tumor microenvironment found this improved prognosis was attributable to cytotoxic CD8+ TILs, while intratumoral CD4+ regulatory T cells (Treg), macrophages and MDSC appear to confer a worse prognosis [13]. Bowtell et al. [14] suggested that a systematic approach for classifying TILs in EOC samples, using a standardized “immunoscore,” could be valuable in determining which patients may benefit from immunotherapies. Recently, an immune gene signature predictive of survival revealed that two of the top five genes are immune genes involved with antigen presentation (*TAP1*) and TIL accumulation (*CXCL9*), providing further support to the concept that immunotherapeutic strategies that boost anti-tumor adaptive immunity will lead to improved prognosis [15].

In one of the largest studies to date, Goode et al. [16] analyzed the frequency of CD8 TILs in more than 5500 patients in relation to progression-free and overall survival. Cases were classified based on the number of CD8+ TILs per high-powered field and categorized as negative (no TILs), low (1–2), moderate (3–19) and high (≥20). The highest predictive value was for patients with HGSOC, where there was a significant positive correlation between the number of TILs and corresponding improvements in survival. Compared to patients with no intratumoral lymphocytes, the median survival of patients with high CD8 TIL counts was almost double (2.8 vs. 5.1 years, respectively). Importantly, this survival predictive effect was not confounded by the volume of residual disease after surgery, suggesting that, in addition to currently validated and widely employed biomarkers such as BRCA1/2 status and progesterone receptor expression, CD8 TILs represent a powerful prognostic biomarker in HGSOC.

Although the majority of cases (55%) in this landmark OTTAC study were HGSOC, the patient cohort also included a significant number of cases (approximately 2400) representing four other, less common ovarian cancer histotypes (endometrioid, mucinous, clear cell and low grade serous). Interestingly, high CD8 TIL levels were also associated with survival among patients with endometrioid and mucinous tumors, but not among patients with clear cell and low-grade serous ovarian cancers. Of note, the endometrioid ovarian cancer cases with the most favorable survival occurred in patients with moderate (not high) CD8 TILs. One possible explanation for this finding (of no added benefit for CD8 TILs above a certain threshold) is that endometrioid tumors with highest CD8 infiltration may be those with mismatch repair deficiency, mirroring findings in endometrial tumors [17].

Collectively, the above studies demonstrate that TILs are present in more than 80% and 70% of HGSOC and endometrioid tumors, respectively, while clear cell and mucinous ovarian tumors show CD8 TILs only in 52% and 51% of cases, respectively. Despite the lower levels of CD8 T cell infiltration, patients with clear cell carcinoma seem to respond much better to immune therapy. Zamarin et al. [18] recently reported that the odds of response to the ipilimumab/nivolumab combination were five times higher in patients with clear cell tumors compared with other ovarian tumor histotypes. Noting that only 12 patients with clear cell tumors were treated in this randomized Phase II trial, the reported results are encouraging, considering that patients in this disease category are particularly challenging to treat. Additionally, these somewhat surprising results point to the gap in our understanding of the mechanisms of response to immune checkpoint blockade therapy, especially in tumors with moderate/low CD8 T cells, where other immune cell subsets and/or non-immune stromal cells may play a central role in controlling tumor immune surveillance.

In exploring the complex immune landscape in EOC and searching for predictive biomarkers of response, the challenge remains to determine which patients will benefit from immunotherapies and to identify how immunotherapy should be incorporated into EOC standard of care, which combines debulking surgery and platinum/taxane chemotherapy. Findings from some of the most important trials featuring immune therapy alone or in combination with chemotherapy are further detailed below.

## 3. Immunomodulatory Properties of Chemotherapeutic Drugs

Platinum derivatives (cisplatin, carboplatin, oxaliplatin) are widely employed, often in association with taxane, and trigger significant therapeutic effects in EOC, where approximately 80% of primary tumors are responsive to platinum-based chemotherapy [19]. Because of their proven clinical efficacy, many immune therapy protocols have been designed to include chemotherapeutic agents. Originally perceived as being immune suppressive, due to the elimination or functional inhibition of anti-tumor immune effectors, studies in more recent years demonstrate that platinum compounds can also act as favorable immune modulators (further reviewed here [20]). Specifically, platinum compounds seem to trigger immunogenic cell death (ICD), characterized by a series of molecular changes, such as increased cell surface expression by dying tumor cells of calreticulin (CRT, release of ATP and HMGB1 (high-mobility group box 1), production of IFN-α (interferon alpha) and secretion of CXCL10 (CXC-chemokine ligand 10) [21]. Collectively, these immune effector molecules act as DAMPs (damage-associated molecular patterns) and promote the activation of antigen presenting cells, augment intracellular antigen processing and presentation and immune priming of the adaptive immune responses.

The capacity of the platinum compounds to trigger ICD varies according to the drug, dose and time of exposure. In EOC patients, carboplatin triggers ICD and does not affect the frequency of blood CD8^+^ T-cells, while increasing their capacity to produce IFN-γ [22]. Using mouse models with different inflammation profiles, we demonstrated that cisplatin triggers MHC class I upregulation and acts as a potent immune modulator in vivo [23]. Based on their properties [24,25], platinum compounds are currently incorporated alongside immune modulators in many chemo-immune therapy combination protocols, some of which are discussed here.

## 4. Monoclonal Antibodies

Monoclonal antibody treatment of cancer has proven to be a successful therapeutic strategy for several malignant tumors. Monoclonal antibodies can exert their function both through direct and indirect mechanisms. Some of these biologic agents directly target tumor driver pathways with the goal of reducing uncontrolled cell proliferation; alternatively, they can bind to surface antigens to enhance immune recognition. Additionally, monoclonal antibodies can exert their effects through indirect mechanisms, by activating antibody-dependent cell-mediated cytotoxicity by NK cells [26,27]. Several antigens have been identified as potential targets of monoclonal antibodies for the therapy of EOC. We review here some of the most heavily investigated targets for monoclonal antibodies, including CA125, human epithelial cell adhesion molecule (EpCAM), folate receptors, VEGF and antibodies targeting immune checkpoints (Figure 1) [28].

### 4.1. CA125

CA125, also known as mucin 16 (MUC16), is a surface glycoprotein and an established marker for monitoring response to treatment and disease relapse [29]. The extensive expression of CA125 in the majority of EOCs makes it an appealing target for monoclonal antibody therapy. Oregovomab, a murine monoclonal antibody targeting CA125, triggers active immunity in the host, as it can induce anti-CA125 T cell immune responses [13,30]. A randomized placebo-controlled phase III trial evaluated the use of oregovomab for maintenance therapy following completion of front-line chemotherapy. While oregovomab showed no clinical benefit in extending the time to disease relapse for patients in first clinical remission, CA125 remains an attractive target (Table 1) [31]. Trials are presently looking to improve oregovomab’s efficacy through combination with immune modulators such as poly IC:LC/Hiltonol (ClinicalTrials.gov Identifier: NCT03162562) or cytotoxic chemotherapies (ClinicalTrials.gov Identifier: NCT04620954) in patients with advanced EOC.

### 4.2. Folate Receptor Alpha

Of the four isoforms of folate receptor, folate receptor alpha (FRα) is commonly targeted in EOC [37,38]. FRα has been reported to be overexpressed in 80–90% of EOCs, but has minimal expression in normal tissues, making it an optimal therapeutic target [39]. Additionally, FRα expression is preserved in recurrent tumors and metastatic foci suggesting a role of FRα in tumor cell survival, progression and chemotherapy resistance [40]. Upregulation of FRα on the surface of tumor cells leads to increased intracellular accumulation of folates, a process that further supports cell division and tumor growth. Furthermore, FRα expression may also induce drug resistance by enhancing the anti-apoptotic capacity of tumor cells [41]. Phase I and phase II trials with farletuzumab, an anti-FRα monoclonal antibody, demonstrated promising results both as a single agent and in combination with chemotherapy [42,43,44]. Unfortunately, recent phase III trials evaluating farletuzumab in combination with platinum/taxane chemotherapy in both platinum-sensitive and -resistant patients have both been terminated after failing to show survival benefit (ClinicalTrials.gov Identifier: NCT00849667, NCT00738699) [32] (Table 1). Efforts to identify an optimal combination regimen and patient population are being made in an ongoing trial evaluating farletuzumab in multiple combinations, in patients with low-CA125, platinum-sensitive ovarian cancer (ClinicalTrials.gov Identifier: NCT02289950).

Mirvetuximab soravtansine (IMGN853) is an anti-FRα antibody-drug conjugate (ADC), consisting of an FRα-binding antibody attached to a highly potent maytansinoid, which induces cell-cycle arrest and death by targeting microtubules [45]. Preliminary data from a phase I trial demonstrated clinical benefit in heavily pretreated patients with EOC and other FRα-positive EOC [37,46]. Following these encouraging results [37], a phase III randomized trial (FORWARD I, NCT02631876) was launched to study a cohort of patients with platinum-resistant disease. FORWARD I investigates the safety and efficacy of IMGN853 compared to the physicians’ choice chemotherapy. The trial enrolled 366 patients with platinum-resistant EOC who had either medium or high levels of FRα expression and a history of 1–3 prior treatment regimens. While the study failed to reach its primary endpoint of progression-free survival, the confirmed overall response rate for IMGN853 was higher than for single-agent chemotherapy (22% vs. 12%, *p*-value 0.015). Furthermore, for patients with high FRα expression, the median progression-free survival was longer for IMGN853 treated patients compared to chemotherapy (4.8 months vs. 3.3 months, HR 0.693, *p*-value 0.049), as well as the confirmed ORR (24% vs. 10%, *p*-value 0.014). IMGN853 was shown to be safe and well-tolerated, with few adverse events. Overall, the FORWARD I study generated promising results for high FRα expressing patients treated with IMGN853 [33] (Table 1). There are several ongoing phase I/II and two studies recently opened phase III trials, evaluating the use of IMGN853 in combination with several different chemotherapy regimens for patients with both platinum-resistant or platinum-sensitive, FRα-positive advanced EOC (NCT04296890, NCT04209855). Results from these studies will potentially provide further support for FRα targeting strategies.

### 4.3. EpCAM

The transmembrane glycoprotein human epithelial cell adhesion molecule (EpCAM) is a one of the most frequently and intensely expressed tumor-associated antigens in a number of malignancies [47]. High levels of EpCAM expression have been associated with worse prognosis in EOC [48]. A phase II/phase III clinical trial compared the efficacy of intraperitoneal anti-EpCAM antibody catumaxomab plus paracentesis to paracentesis alone in 258 patients with recurrent adenocarcinoma. This study found that while catumaxomab delayed ascites re-accumulation, it did not impact disease progression or overall survival [34]. Recent results from a phase II trial demonstrate that IP administration of catumaxomab followed by systemic chemotherapy in patients with gastric cancer-associated peritoneal carcinomatosis is feasible and tolerable. Although the primary endpoint (microscopic complete remission) was not reached, the results support integration of IP immunotherapy into multimodal therapeutic approaches [49]. Catumaxomab use in the United States remains limited to clinical trials, but in April 2009, catumaxomab was approved in the European Union for treatment of malignant ascites in patients with EpCAM-positive carcinomas where standard therapy is not available or is no longer feasible.

### 4.4. VEGF

Vascular endothelial growth factor A (VEGF) is a prominent cytokine which promotes angiogenesis and a key target of anti-cancer therapies. VEGF plays a major role not only in controlling blood vessel formation, tumor growth, invasion and metastasis but also in modulating tumor-induced immunosuppression. This effect occurs by enhancing the mobilization of Tregs and inducing M2 polarization of tumor-associated macrophages. VEGF can also activate myeloid derived suppressor cells (MDSC), which in turn produce more VEGF. Furthermore, VEGF inhibits dendritic cell (DC) maturation, thus reducing priming of CD8+ cells to tumor antigens.

To reduce these VEGF-induced immune suppressive effects, inhibition of VEGF activity leads to reduced Tregs, TAMs and MDSC, increased antigen presentation by DCs, enhanced T cell activation in the priming phase and improved TIL accumulation [50,51]. VEGF inhibitors, therefore, have the potential to promote an immunostimulatory tumor microenvironment [52].

Bevacizumab is a humanized anti-VEGF monoclonal IgG antibody approved for use in combination with chemotherapy for the treatment of various cancer types, including EOC. GOG-0218 is a phase III, randomized trial of bevacizumab in women with newly diagnosed, incompletely resected ovarian, fallopian tube or primary peritoneal carcinoma. The cohort of 1873 women was randomized 1:1:1 to chemotherapy (six 21-day cycles of intravenous carboplatin and paclitaxel) versus chemotherapy plus concurrent bevacizumab (cycles 2 to 6) versus chemotherapy plus concurrent and maintenance bevacizumab (cycles 2 to 22). No survival differences were observed for patients who received bevacizumab compared with chemotherapy alone [35]. However, a significant survival benefit was observed for patients with stage IV disease treated with upfront and maintenance bevacizumab. Additionally, patients with BRCA1/2-mutated tumors had a statistically significant 38% reduction in the survival hazard, as compared with BRCA wild-type cases. Similarly, a statistically significant 35% reduction in the hazard occurred in patients with non-BRCA homologous recombination repair (HRR) deficiency, providing support for combination with PARP inhibitors (PARPi).

Bevacizumab and olaparib (a PARPi) combination was recently tested in a phase 3 randomized trial as a potential maintenance therapy for EOC, regardless of the presence of a BRCA mutation (PAOLA-1, NCT02477644). A total of 806 patients with newly diagnosed, advanced, high grade EOC were studied for a median follow-up time of 22.9 months. The median progression free survival with olaparib plus bevacizumab was 22.1 months compared to 16.6 months with placebo plus bevacizumab. Furthermore, in patients with tumors positive for homologous-recombination deficiency (HRD), including tumors with BRCA mutations, median progression free survival was 37.2 months for olaparib plus bevacizumab compared to 17.7 months for the placebo group. For HRD-positive tumors with no BRCA mutations, median progression-free survival was 28.1 vs. 16.6 months for the olaparib group vs. placebo group. These results indicate that the combination of anti-VEGF-A therapy with PARPi is effective as a first-line maintenance therapy for patients with high-grade, advanced EOC, and provides a substantial progression-free survival benefit, regardless of the presence of a BRCA mutation [36]. Studies featuring promising combinations of bevacizumab and immune checkpoint blockade are discussed below.

### 4.5. Immune Checkpoint Blocking Antibodies

An important group of monoclonal antibodies currently under investigation for therapy of various solid tumors, including EOC, are inhibitory receptor blocking antibodies called immune checkpoint inhibitors. Immune checkpoints represent inhibitory pathways comprising a series of receptor-ligand interactions, normally used to maintain self-tolerance. Tumors co-opt these pathways as a mechanism of immune resistance, particularly to evade tumor-specific T cells. Several receptor/ligand interactions have been described to date as part of the immune checkpoint pathway, and the list is rapidly expanding [53]. We discuss here only the most developed therapies, using immune checkpoint blockade (ICB), currently tested in clinical trials for EOC [54,55,56].

### 4.6. CTLA-4

Cytotoxic T-lymphocyte-associated protein 4 (CTLA-4) acts as a brake on the surface of T cells, leading researchers to explore whether blocking CTLA-4 might augment anti-tumor T cell-mediated immunity. Early studies in metastatic melanoma show that treatment with anti-CTLA-4 blocking antibody (ipilimumab) confers significant therapeutic benefit, leading to the first FDA approval of ipilimumab for melanoma patients. Antibodies targeting CTLA-4 maintain T cell activation by blocking inhibitory signaling normally enacted when CTLA-4 binds to CD80 and CD86 on the surface of antigen-presenting cells [57]. Even though the exact mechanism of action for ipilimumab has not been fully described, evidence to date suggest that it has dual activity, by promoting effector CD8 T cells while driving the depletion of CTLA4^hi^ Tregs (Figure 1). In EOC, the benefit of CTLA-4 blockade remains under investigation. In a phase I study including 11 patients with recurrent EOC, all previously vaccinated with granulocyte macrophage colony-stimulating factor (GM-CSF), one patient had an objective response that was durable over four years and three patients had stable disease. Of note, two patients did experience grade 3 gastro-intestinal toxicity after a single dose [58]. A phase II trial of single agent ipilimumab was performed in 40 patients with platinum-sensitive EOC. Unfortunately, 38 (95%) of patients did not complete the induction phase (10 mg/kg ipilimumab every 3 weeks × 4 doses) due to disease progression (14, 35%), drug toxicity (17, 42.5%), death (1, 2.5%) or other/unreported events (6, 15%) [59] (Table 2).

In addition to monotherapy toxicity, emerging evidence suggests that clinical responses in EOC are less than those seen with CTLA-4 blockade in melanoma, pointing to the need for alternative approaches. One potential efficacious regimen currently evaluates CTLA-4 blockade in combination with PARP inhibition for patients with BRCA-deficient EOC (NCT02571725).

### 4.7. PD-1/PD-L1

Immune cells, and particularly activated T lymphocytes, express on their cell surface a molecule called programmed death-1 (PD-1). When PD-1 binds to its ligand, PD-L1, T cells are rendered inactive against their target. Tumor cells upregulate PD-L1 as a mechanism to protect themselves against the immune attack (Figure 1). Like CTLA-4, PD-1 and PD-L1 are part of the immune checkpoint pathway and blocking antibodies that prevent PD-1/PD-L1 interactions are under ongoing investigations in numerous cancer types, including EOC. The first study to demonstrate a benefit of PD-1 blockade was a phase I study of anti-PD-L1 antibody in patients with advanced solid tumors, which included 17 EOC patients. In this group, one patient had a partial response and three patients had stable disease [64]. These results provided a rationale for further investigation of PD-1/PD-L1 inhibition with other agents including nivolumab (anti-PD-1 antibody), pembrolizumab (anti-PD-1) and avelumab (anti-PD-L1). Preliminary analysis from a phase IB trial of avelumab included 75 patients with recurrent platinum-resistant EOC, and showed 8 patients with partial response and 33 with stable disease, giving a disease control rate of 54.7% [65]. A phase IB trial of pembrolizumab (anti-PD1-antibody) included 26 patients with advanced EOC with tumors confirmed to express PD-L1. Of this group, one patient had complete response, two patients had partial response and six patients had stable disease [66]. Finally, in a trial of nivolumab (anti-PD-1 antibody), 20 patients were treated, with 10 patients in each dose cohort. In the low-dose cohort one patient had partial response and four had stable disease, and in the high-dose cohort, two patients had complete response and two had stable disease [60] (Table 2). Across these studies, immune checkpoint inhibitors were generally well-tolerated.

The phase 1b of the JAVELIN Solid Tumor trial recruited 125 patients with recurrent or refractory EOC and disease progression after platinum-based chemotherapy. Patients received avelumab 10 mg/kg, every 2 weeks until disease progression, unacceptable toxic effects or withdrawal from the study. An objective response occurred in 12 patients, including a complete response in 1 patient (0.8%) and a partial response in 11 patients (8.8%). The 1-year progression-free survival rate was 10.2% (95% CI, 5.4–16.7%) and median overall survival was 11.2 months (95% CI, 8.7–15.4 months). Despite the encouraging response and survival findings, the results of this study reveal modest clinical efficacy of avelumab when administered as monotherapy in this patient population [67].

The largest study of single-agent immune checkpoint in EOC is KEYNOTE-100 (NCT02674061), a phase II trial that examined the clinical activity of single-agent pembrolizumab (anti-PD-1) in patients with recurrent disease. Two patient cohorts were enrolled: cohort A enrolled less heavily pretreated patients (defined as one to three prior lines of treatment), while cohort B enrolled patients who were more heavily pretreated with platinum-based chemotherapy (defined as four to six prior lines of treatment). Cohort A enrolled 285 patients, 100 of whom served as a PD-L1 biomarker training set. Cohort B enrolled 91 patients. Results showed a 7.4% ORR for cohort A and 9.9% for cohort B, pointing to the fact that pretreatment did not seem to affect the response rate to pembrolizumab. Additionally, the PD-L1 biomarker analysis indicated that patients with higher PD-L1 expression had a better response to treatment [61] (Table 2). The overall low ORR reported in this trial demonstrates that, in contrast to the more encouraging results from other cancers (such as melanoma, lung and head and neck cancers), single agent pembrolizumab is effective in only a very small subset of the EOC patient population and that more effective combination therapies are needed.

Additional trials are currently underway to test the checkpoint blockade in combination with other therapies, such as chemotherapy, PARPi, anti-vascular agents and other biologic agents that can synergize the effectiveness of checkpoint blockade in the upfront or recurrent disease settings. Several large phase III trials test new chemo-immunotherapy combinations and have started accruals in the past few years. JAVELIN Ovarian 100 is an international, multi-center, randomized phase III clinical trial that examined the efficacy of anti-PD-L1 antibody avelumab in combination with and/or following platinum-based chemotherapy in newly diagnosed EOC patients (NCT02718417). The study included three arms: chemotherapy (carboplatin/paclitaxel) followed by observation; chemotherapy followed by avelumab maintenance; chemotherapy plus avelumab followed by avelumab maintenance. Unfortunately, the study failed to meet its primary endpoint of improving progression-free survival in EOC patients and was discontinued [62] (Table 2).

JAVELIN Ovarian 200 is an international, multi-center phase III clinical trial which enrolled 566 patients with platinum-resistant/refractory EOC (NCT02580058) [68]. This study examined whether avelumab (anti-PD-L1) given alone or in combination with pegylated doxorubicin (PLD) is superior to PLD alone in prolonging overall survival in patients with platinum-resistant/platinum-refractory ovarian cancer. Patients in this trial were randomized to one of three arms: PLD alone, PLD plus avelumab or avelumab alone (44). While results showed no improvement in overall survival nor progression-free survival in the overall population of trial patients treated with PLD plus avelumab compared to PLD, an exploratory biomarker analysis revealed that patients expressing specific biomarkers may receive some benefit from combination therapy. Patients receiving PLD plus avelumab who were PD-L1-positive had an ORR of 18.5% compared to a 3.4% ORR for PD-L1-negative patients. The difference in response rate is small but could indicate that PD-L1 expression may play a part in predicting the clinical activity of combination therapy using avelumab compared to chemotherapy alone [63] (Table 2).

Two additional Phase III ovarian cancer trials will assess the efficacy of atezolizumab (anti-PD-L1) in combination with bevacizumab and chemotherapy. ATALANTE trial (ClinicalTrials.gov Identifier: NCT02891824) will assess the efficacy of atezolizumab in combination with bevacizumab plus platinum-based chemotherapy in patients with platinum-sensitive disease who have late relapse (platinum-free interval >6 months). NCT03038100 (IMagyn050) trial will test the activity of atezolizumab in combination with bevacizumab and paclitaxel/carboplatin in patients with newly diagnosed ovarian cancer. Collectively, these combination trials have the potential to advance our ability to select the optimal chemo regimen, identify the correct sequencing, timing, and dose and to efficiently manage concurrent toxicities.

The combination of ICB with the inhibition of poly-ADP ribose polymerase (PARP) represents another promising therapeutic venue in EOC [69,70]. Inhibition of PARP (PARPi) leads to accumulation of DNA breaks and induction of apoptosis in tumor cells carrying defects in homologous recombination or double stranded DNA repair function. This “synthetic lethality” is typically seen in BRCA1/2 mutated EOC, but other subtypes may also benefit. Because EOC with BRCA mutations as well as damaging mutations of homologous recombination repair genes are associated with high neoantigen formation and T cell neoepitope presentation, these cancers represent a suitable target for immunotherapy. ATHENA is a phase III randomized trial that evaluates rucaparib (a PARPi) and nivolumab (anti-PD-1) as maintenance treatment in patients responding to front-line platinum-based chemotherapy (ClinicalTrials.gov Identifier: NCT03522246). The target enrollment of 1000 patients with advanced EOC, fallopian tube or primary peritoneal cancer makes this one of the premier studies currently testing the efficacy of ICB/PARPi combination in EOC patients [71].

TOPACIO/KEYNOTE-162 trial (NCT02657889) was an open-label, single-arm phase I/II integrated study which evaluated the clinical activity and safety of pembrolizumab in combination with niraparib (PARPi) in platinum-resistant, recurrent OC (ROC). The results of this study showed this combination to be tolerable and indicated no new toxicity or safety concerns. Importantly, antitumor activity was also shown for this population of patients, including those with BRCA-wild type (wt) or non-HRD disease. The study reported an overall response rate (ORR) of 18% (90% CI, 11–29%) and a disease control rate of 65% (90% CI, 54–75%). Three patients (5%) developed confirmed complete responses, 8 (13%) partial responses, 28 (47%) had stable disease and 20 (33%) progressed under treatment [72]. Although the predefined statistical criteria for this study were not met, the reported ORR for Pembrolizumab/niraparib in patients with platinum-resistant/refractory recurrent disease, especially in the BRCA-wt and non-HRD patient populations that are unresponsive to other treatments, is encouraging.

In follow-up studies on the TOPACIO trial, Färkkilä et al. [73] recently examined new mechanistic determinants of response to the niraparib/ pembrolizumab combination [72]. In addition to the tumor homologous recombination DNA repair and mutational status, responding patients also had a positive immune score suggestive of interferon primed, exhausted CD8 + TILs. Furthermore, spatial profiling using multiplex imaging of interactions between immune cell subpopulations in the TME of responding cases revealed that tumor tissue areas with the highest scores for exhausted CD8 + T-cells also involved proximity to PD-L1 + cancer cells, macrophages and DCs [73]. By using tumor tissue profiling with single cell imaging resolution capability, this study suggests that spatial interactions between tumor cells and various immune cell subsets with different functional states may serve as biomarkers of response to immunotherapy, and that incorporation of advanced multiplexed imaging may greatly impact biomarker discovery for immuno-oncology and provide new modalities for patient stratification [73].

JAVELIN PARP Medley is an ongoing phase1b/2 trial which is enrolling a cohort of patients with recurrent platinum-sensitive ovarian cancer who will be treated with avelumab plus talazoparib (ClinicalTrials.gov Identifier: NCT03330405). Results from this and other ongoing trials will further define the role of PARPi/checkpoint inhibitors combination within the treatment of EOC.

We recently initiated at our own institution a phase II trial in which patients with recurrent EOC receive a total of six treatment cycles, at 3-week intervals (ClinicalTrials.gov Identifier: NCT03734692). The study uses IP neoadjuvant approach (IP cisplatin) and IV infusion of anti-PD-1 (pembrolizumab). In addition, patients also receive IP rintatolimod (a toll-like receptor 3, TLR3 agonist). Cytoreduction (removal of residual tumor) will occur approximately 4 weeks after the fourth treatment cycle. Post-surgery, the patients will receive two additional courses of the same, three-drug chemo-immunotherapy regimen. This trial builds on our recent results demonstrating the immune adjuvant effect of cisplatin, which can be further enhanced by immune modulators such as rintatolimod and ICB [23,74].

A rapidly growing number of trials incorporates anti-PD-1/PD-L1 blockade in various treatment protocols for advanced EOC. Results from these studies will define how to best fit immune checkpoint inhibitors into the standard treatment of EOC. Furthermore, they will hopefully unravel new mechanisms of response and identify outcome-predicting biomarkers. 

## 5. Vaccines

Overall, epithelial ovarian tumors are considered immunogenic and can trigger spontaneous T cell and B cell/antibody responses against the tumor [10]. Patients with high numbers of TIL and measurable tumor-specific cellular and/or humoral immunity have better overall survival, prompting the hypothesis that induction of robust antigen specific T cells and/or antibody responses through active immunization might improve the clinical outcome in EOC patients [75,76,77]. The concept of active immunization through cancer vaccines that can trigger robust anti-tumor immune effectors (Figure 1) has been historically tested in EOC for more than two decades. However, the results from numerous early phase clinical trials, with various vaccine formulations (recently reviewed by Martin Lluesma et al. [78] have been rather modest. Despite the fact that vaccine-induced responses can be identified in some of the vaccinated recipients, such responses are of low amplitude and infrequently found [79]. The majority of patients do not show improved clinical outcome, most likely due to immune escape mechanisms [13,80]. Recent advances with ICB in melanoma, lung and head and neck cancers demonstrate that tumor-specific cytotoxic T cell responses are restored during treatment, often with therapeutic benefits (reviewed in [81]). In EOC, efficacy of ICB alone is modest, raising the potential need for combination therapy [64,82]. Given that vaccines can increase the pool of effector T cells, ICB/vaccine combinations may have synergistic effects, providing the rationale for further developments on EOC vaccines [56,83,84,85].

The classical composition of a cancer vaccine comprises an antigenic fraction and an adjuvant. To date, most antigens tested are tumor associated antigens (TAA). Among the TAA frequently employed in OC are WT1, MUC1, NY-ESO-1, folate receptor, Her-2/neu and p53 (Figure 1). These antigens are combined with adjuvants, most often Montanide, GM-CSF or poly ICLC [86,87]. We review here some of the vaccination approaches based on EOC TAA and focus primarily on the new vaccine/ICB combination currently tested in early phase clinical trials. Although results from the trials described below are currently pending, the novel combination therapies tested in these trials have increased the immune therapeutic potential.

### 5.1. WT1

Wilms tumor 1 (WT1) is a transcription factor with well-defined oncogenic properties [88]. WT1 is overexpressed in many solid tumors, including 93% of serous OC and has been ranked as the most promising cancer antigen, well suited for vaccine development [89]. To date, WT1 vaccines have been administered to patients with ovarian, breast, lung and renal cancers and glioblastoma [90,91,92]. Successful WT1 peptide vaccination for childhood malignancies has also been reported [93]. Based on encouraging evidence (albeit from small studies) showing measurable WT1-specific anti-tumor CD8+ T cell responses [94,95], a WT1 peptide vaccine using Montanide and GM-CSF as adjuvants is currently being explored in a phase I study in patients with recurrent OC who are in second or later remission. The vaccine is administered in combination with anti-PD-1 antibody (Nivolumab) and represents the first WT1 vaccine /ICB combination to be explored in EOC (NCT02737787).

### 5.2. MUC1

Mucin1 (MUC1) is an oncogene overexpressed by most adenocarcinomas (of the breast, uterus, prostate, stomach, colon, pancreas). These include all EOC, regardless of histology [96]. Based on its wide expression profile, antigenic and immunogenic properties first demonstrated in EOC, MUC1 ranks second, after WT1, on the list of prioritized antigens for vaccine development [89]. MUC1 vaccines have been employed in numerous forms, including short (MHC-I-restricted) or large (MHCII-restricted) MUC1 peptides, or MUC1 encoding mRNA or DNA [97,98,99]. Presently, a phase I trial is testing an adenoviral vector encoding a fusion protein in which MUC1 epithelial antigen is attached to the CD40 ligand (NCT02140996). In addition to EOC patients, this non-randomized open label dose escalation trial also includes men or women with metastatic or recurrent epithelial cancers of the lung, breast, prostate and colon. No MUC1/ICB vaccination protocols are currently being tested in the clinic. However, preclinical studies from our group using MUC1 transgenic mice with MUC1-expressing tumors demonstrate efficacy of MUC1 peptide vaccines and PD-L1 blockade as single therapies, respectively [100,101]. These preclinical results provide a strong rationale for the future development of MUC1 vaccine/ICB combinations.

### 5.3. NY-ESO-1

A member of the family of cancer testes antigens, NY-ESO-1 is overexpressed in a subset of epithelial EOC and can induce measurable CD8 and antibody responses [102,103,104]. Results from a phase II trial revealed that patients with a measurable immune response after vaccination with NY-ESO-1 peptide experience prolonged survival [104]. An improved vaccine formulation, comprising NY-ESO-1 fusion protein (CDX-1401) in combination with anti-DEC-205 mAb, adjuvant poly-ICLC (a synthetic complex of carboxymethylcellulose, polyinosinic-polycytidylic acid and poly-L-lysine double-stranded RNA) and indoleamine 2,3-dioxygenase (IDO1) inhibitor INCB024360, is currently being tested in a Phase I/IIb study of OC patients in remission (NCT02166905). IDO1 enzyme is involved in the catabolism of tryptophan and its overexpression in the tumor microenvironment suppresses the cytolytic activity of CD8 and NK cells and promotes Treg function [105]. Similar to PD-1/PD-L1, CTLA-4, TIM-3 and LAG-3, IDO1 is also considered an inhibitory immune checkpoint molecule [105,106] and the NY-ESO-1/IDO1 inhibitor is one of the emerging peptide vaccine/ICB combination with potentially increased efficacy [107].

### 5.4. Folate Receptor Alpha

As discussed above, FRα is a target under investigation for EOC. Vaccines using FRα protein or peptides trigger anti-FRα antibodies and CD8 T cell responses, as reported in several clinical trials in EOC as well as in other FRα positive solid tumors [108,109]. In NCT02764333, a new multi-epitope FRα vaccine/ICB (anti-PD-L1) combination is tested in a phase II clinical trial in patients with platinum-resistant disease. Block et al. [110] recently reported results from a single arm, open label phase I trial that tested five FRα-derived epitopes pulsed onto DCs generated using a Th17-inducing protocol (ClinicalTrials.gov Identifier: NCT02111941). The vaccine, in combination with granulocyte-macrophage colony-stimulating factor (GM-CSF), was administered to EOC patients in first remission [40]. The Th-17-inducing vaccine was well-tolerated and highly immunogenic, leading to augmented immunity to FRα in over 90% of patients, as demonstrated by interferon gamma (IFN-γ) ELISPOT testing [40]. Notably, although the study was not powered to assess clinical outcomes, the median recurrence-free survival in 10 patients at first remission was significant, at 528 days [110]. While these encouraging results point to Th17-inducing DC vaccination as being well-tolerated, and highly immunogenic, the clinical benefit of this approach in EOC needs to be further explored.

### 5.5. TP53

The tumor suppressor p53 is the most frequently mutated gene in EOC. In high-grade serous ovarian cancer, the most common EOC histotype, *TP53* mutations are found in in over 96% of cases, and can cause either loss of protein expression or loss of wild-type p53 function, with or without increased accumulation of defective p53 protein [111]. In humans, spontaneous cellular and antibody mediated immune responses targeting p53 protein have been observed and several MHC class I and II restricted epitopes have been identified [112,113,114,115]. An ongoing phase I clinical trial currently tests the hypothesis that vaccination of patients with recurrent tumors positive for defective p53 protein increases p53-specific anti-tumor immunity. This protocol employs a vaccinia virus ankara vaccine expressing p53, together with gemcitabine hydrochloride (ClinicalTrials.gov Identifier: NCT02275039).

### 5.6. Whole Tumor Cell Lysates

As an alternative to defined tumor antigens, whole lysates can also be used for vaccination, and have been generally loaded onto DCs [116]. Chiang and colleagues [117] recently tested the therapeutic efficacy of autologous DCs loaded with hypochlorous (HOCL)-oxidized autologous tumor lysate [117] (ClinicalTrials.gov Identifier: NCT01132014) administered to EOC patients with stage III/IV disease. In addition to the vaccine, patients received low-dose cyclophosphamide and therapeutic doses of acetyl salicylic acid, included for its potential to inhibit tumor VEGF, lower prostaglandin production and attenuate Treg activity. Preliminary results suggest that vaccination is effective and can induce robust anti-tumor responses. In a follow-up trial (ClinicalTrials.gov Identifier: NCT01312376) the investigators collect DC vaccine-primed T cells and prepare them for adoptive T cell transfer following ex vivo anti-CD3/CD28 expansion. Importantly, recent results demonstrate that vaccine primed T cells express the cognate receptors specific for lymphocyte recruiting chemokines (such as CCL-2, CCL-4 and CCL-5, as well as CXCL-10, CXCL-12 and CXCL-16) released by the majority of tumors [118,119]. Overall, these findings demonstrate that the ovarian tumor microenvironment is conducive to homing of vaccine-primed, adoptively transferred T cells and supports the development of future trials combining vaccines and adoptive transfer therapy.

### 5.7. Neoantigens

Tumors with high mutational burden express mutated proteins, called neoantigens. Fragments from these tumor-specific antigens (neoepitopes) are processed by antigen-presenting cells and presented to the patient’s own T cells. In the right context of immune co-stimulation, T cells will recognize these fragments as “new,” and will mount a more vigorous yet targeted, tumor-specific immune response. This paradigm has been proven in mutated tumors such as melanoma, lung and renal cancer, where the “enriched,” neoepitope-specific T cells drive the response to immune checkpoint blockade.

However, in EOC, the genomic profiling data offer a contrasting picture. Regardless of the histologic type, very few ovarian tumor cases demonstrate a high mutational burden or high rates of mismatch repair (4). Excluding the high rate of *TP53* mutations (found in more than 95% of HGSOC), the defining trait of EOC is the aberrant gene copy number; thus, it is unlikely that ovarian tumors display a high frequency of neoantigens [111].

Nevertheless, despite the low rate of neoantigen positivity, solid evidence of mutation-reactive T cells infiltrating ovarian tumor masses currently exists. Neo-epitope specific CD8+ T cells were detected in ~90% of patients evaluated by Bobisse et al. [120], and mutation-reactive T cells were generated in patients vaccinated with tumor cell lysate [121].

Using a complex methodology, studies from Rosenberg et al. demonstrated that T cell receptors (TCRs) recognizing neoepitopes (including those spanning the *TP53* “hotspot” mutations) are present in some ovarian cancer patients and could be employed as gene therapy [122]. Neo-epitope validation in TILs offers encouraging evidence for low-mutational burden tumors such as ovarian cancer and extends opportunities for mutanome-based personalized immunotherapies. One should acknowledge that while feasible, such personalized approaches rely on the availability of highly sensitive, time consuming and costly methodologies, raising technical challenges about their applicability to the wider ovarian cancer patient population.

## 6. Cellular Immunotherapy

Adoptive cell therapy, also known as cellular immunotherapy, is a treatment modality that employs the patients’ own immune cells to eliminate cancer. Some of these approaches involve isolation, ex vivo expansion and reinfusion of TILs, whereas others involve genetically engineered solutions (via gene therapy) to enhance the immune cells’ cancer-fighting capabilities.

### 6.1. TIL Therapy

Clinical efficacy of adoptive transfer of expanded TILs was first demonstrated more than three decades ago, in patients with metastatic melanoma [123,124]. ACT was also tested in EOC in the 1990s and showed promise for metastatic disease [125,126]. A pilot trial conducted more recently examined the prospect of combining TIL ACT with immune checkpoint inhibitors for HGSC. A study of six patients with late-stage metastatic HGSC were treated with ipilimumab, underwent surgery to remove TILs and were infused with ex vivo expanded tumor-infiltrating lymphocytes (REP-TILs), IL-2 and nivolumab. One patient achieved a partial response, while the remaining five patients had stable disease for up to 12 months following treatment [127]. Worldwide, there are currently several early-stage clinical investigations of expanded autologous TILs, (such as OVACURE, ClinicalTrials.gov Identifier: NCT04072263, NCT03412526), which aim to further establish the efficacy of this approach.

### 6.2. TCR Engineered Peripheral Blood Mononuclear Cells (PBMC)

Encouraging results from vaccination studies using TAA have demonstrated that vaccine-induced CD8 T cells are capable to recognize and eliminate tumor cells in an antigen-specific, MHC-restricted manner [128]. These findings have subsequently led to the concept that isolation of T cell receptors (TCR) from the most effective anti-tumor CD8 T cells from patients, and can be employed for gene therapy-based treatments. Successful results from early preclinical studies testing this model have led to current clinical applications in EOC, using retroviral vectors encoding for several TCR, specific for TAA such as NY-ESO-1, WT1 or p53 [129,130,131,132]. The NY-ESO-1 TCR has good safety profile and has so far demonstrated efficacy in melanoma and synovial sarcoma [130]. Results from ongoing studies with NY-ESO-1-specific, HLA-A2-restricted TCR in EOC (ClinicalTrials.gov Identifier: NCT01567891) are pending.

MAGE-A3, a member of the melanoma-associated antigen family, is a TAA overexpressed by several solid tumors, including EOC. An ongoing phase I/II clinical trial explores TCR immunotherapy targeting MAGE-A3 in HLA-DP0401 positive patients with metastatic cancer (NCT02111850). Infusion of the TCR-gene engineered lymphocytes is accompanied by the administration of Aldesleukin (IL-2). The safety profile of this approach remains to be established.

ErbB-2 (or HER2) is a protein kinase with oncogenic properties, and an attractive candidate antigen for targeted TCR-based cancer immunotherapy. Studies from Lanitis et al. [133] demonstrated that a TCR specific for ErbB-2 peptide 369–377, isolated from an HLA-A2+ patient vaccinated with ErbB-2 peptide-pulsed DC vaccine, holds promise for targeting EOC.

Despite the clear progress with generating genetically engineered, antigen-specific TCRs, there are several limitations that may thwart their clinical efficacy, such as MHC loss and defects in antigen processing by tumor cells. As an alternative, T cells can be transduced to express a more versatile, non-MHC restricted chimeric antigen receptor, specific for surface TAA.

### 6.3. Chimeric Antigen Receptors

Chimeric antigen receptors (CARs) represent novel constructs that carry the tumor antigen recognition domain of a monoclonal antibody coupled with the intracellular signaling properties of a TCR (Figure 1). Genetically engineered CAR T cells are highly versatile in the way that they recognize TAA in a non-MHC restricted manner and are cytotoxic due to the TCR-mediated effector mechanisms. The process of CAR-T administration to patients requires leukapheresis followed by the isolation of autologous T cells, which are then genetically engineered to express a modified T cell receptor. CAR-T cells are subsequently infused into patients, after a lymphodepleting regimen [62,134]. Despite their promising efficacy, CAR-T cell infusions can lead to toxicities in a significant portion of patients [59,135]. We focus here on the most promising CARs, currently tested in ongoing trials for EOC (further reviewed here [136]).

#### 6.3.1. CAR T Cells Targeting MUC1

Mucin 16 (MUC16, also known as CA125) is an ovarian cancer antigen over-expressed by a majority of EOC [137,138]. The MUC16 is a glycoprotein composed of a large extracellular domain with multiple repeat sequences that can be cleaved and released in the circulation, and a cytoplasmic domain comprising a short, non-repeated extracellular portion (MUC16^ecto^), a transmembrane region and cytosolic tail that can be phosphorylated. Koneru et al. [139] recently reported the design of a Phase I clinical trial in which T cells engineered to express CAR specific to MUC16^ecto^ (and armored with the ability to secrete IL-12 as well as with a safety elimination gene) are injected IV and IP in patients with recurrent platinum-resistant EOC. While this T cell therapeutic approach shows in vivo efficacy, results from the trial (ClinicalTrials.gov Identifier: NCT02498912) are pending [139].

#### 6.3.2. CAR T Cells Targeting Mesothelin

Mesothelin is a tumor antigen expressed by EOC, as well as by other solid tumor types [140,141,142]. In one of the ongoing trials, patients with metastatic cancer positive for mesothelin (including mesothelioma, ovarian, cervical, lung and pancreatic tumors) undergo lymphodepleting conditioning with cyclophosphamide and fludarabine, followed by intravenous infusion of ex vivo engineered, anti-mesothelin CAR expressing T cells plus low dose IV Interleukin-2 (ClinicalTrials.gov Identifier: NCT01583686).

Development of mRNA CAR transfected peripheral blood lymphocytes recognizing human mesothelin (CARMA-hMeso) has been recently reported, using a simplified manufacturing approach [143]. CARMA is a streamlined, cGMP-compliant strategy for manufacturing PBMCs transfected with antigen-specific mRNA CAR without prior expansion or activation. Preclinical animal studies demonstrate that IP injection of CARMA-hMeso triggers dose-dependent inhibition of tumor growth and improved survival of mice, with no significant off-target toxicities [143]. These results support the evaluation of IP CARMA-hMeso in patients with platinum-resistant EOC.

CAR T cells targeting other cell surface antigens have shown preclinical efficacy either alone or in combination with ICB [144,145]. Recent studies have focused on aberrantly glycosylated cell surface proteins expressed at higher levels by tumor cells, making them amenable CAR targets. Tumor-associated glycoprotein 72 (TAG72) is the sialyl-Tn antigen present on multiple O-glycoproteins expressed by several cancer types, including EOC. Murad et al. [146] recently developed a humanized TAG72-specific CAR containing a 4-1BB intracellular co-stimulatory signaling domain. Preclinical results demonstrate that these glycoepitope-specific CAR T cells exert potent antigen-dependent cytotoxicity against multiple TAG72+ ovarian cancer targets, which supports their translation into the clinic [146]. CAR constructs that selectively bind MUC1 that carries the Tn or sialyl (S)Tn glycans have also been developed, further supporting the notion that aberrantly glycosylated antigens represent a novel class of cancer therapeutic targets [147].

T cells engineered with CAR specific for HER2 (ClinicalTrials.gov Identifier: NCT01935843) and epidermal growth factor receptor (EGFR, ClinicalTrials.gov Identifier: NCT01869166) are now tested outside of the US, in patients with solid tumors (including ovarian tumors) positive for HER2 and EGFR, respectively.

#### 6.3.3. NKG2D CAR-T Cells

NK cells can recognize different types of tumor cells through various cell surface receptors. In addition to scFv regions or TCR binding domains, NK cell receptors have also been used in the development of CARs. NK cell receptors bind to ligands that can be expressed by a variety of solid and hematologic tumors, as well as by immune suppressive cells such as Tregs and MDSCs, providing attractive candidates for therapy. One of the most studied is the NK cell receptor NKG2D and its ligands, which are selectively expressed by tumor cells [148]. In addition to NK cells, NKG2D receptors can also be found on CD8+ T cells, and a subset of CD4+ T cells, NKT cells and γδ T cells [149,150]. An ongoing multi-national dose escalation phase I trial called THINK (THerapeutic Immunotherapy with NKR-2) will test an infusion of NKG2D-CAR T cells (NKR-2 cells) administered every 2 weeks for a total of 3 infusions within 4 weeks. In addition to EOC, the study cohort includes four refractory solid tumors (colorectal, bladder, triple-negative breast and pancreatic cancers) and two hematological tumor types (acute myeloid leukemia and multiple myeloma, NCT03018405).

## 7. Immune Therapy with Oncolytic Viruses

Oncolytic virus therapy is based on the concept that modified viruses can infect tumor cells, which causes them to self-destruct and generate immunogenic cell death. More than 20 viruses with tropism for tumor cells have been tested as treatments for various malignancies. Of these, 11 have shown preclinical efficacy in EOC [151]. For the vast majority of cases, recurrent ovarian cancer remains confined in the peritoneal cavity, providing an opportunity for loco-regional administration of novel therapeutics, including immune virotherapy agents [152]. An ongoing randomized phase II trial testing an attenuated measles virus (MV) genetically enhanced to express the thyroidal sodium symporter gene (NIS) is currently enrolling patients with ovarian, fallopian tube or peritoneal cancer (NCT02364713). This randomized phase II trial compares MV-NIS to investigator’s choice chemotherapy. The choice of an engineered MV is based on the fact that ovarian tumor cells express high levels of MV receptors CD46 and nectin-4 and that MV may be able to kill tumor cells without damaging normal cells [153].

The rationale for NIS, a membrane ion channel normally found in thyroid follicular cells for iodide uptake, postulates that upon infection with MV-NIS, NIS-expressing ovarian tumor cells show augmented uptake of radioiodine isotopes (^125^I), conferring an additional therapeutic benefit [154]. Despite the challenges ahead, oncolytic virotherapy has the potential to improve clinical outcomes for EOC patients.

## 8. Type I Interferon Production Agonists

A recent classification of tumors as either T-cell inflamed or ‘hot tumors,’ or non-T cell inflamed or ‘cold tumors,’ has shaped the present understanding of chemoresistance in EOC and other cancer types [155]. Chemoresistance is typically associated with ‘cold tumors,’ which show decreased expression of type 1 interferon (IFN) genes and a lower density of CD8^+^ TILs. As such, the microenvironments of these tumors are not well-equipped to recruit immune cells and initiate antitumor immune responses. Therefore, new approaches that can stimulate the expression of type I IFN genes and enhance CD8+ cytotoxic T cell recruitment may become attractive options to combat chemoresistant ovarian tumors and to increase response to immunotherapy.

### STING Agonists

Stimulator of interferon genes (STING) is an endoplasmic reticulum transmembrane protein that plays an important role in innate immunity by mediating IFN1. After sensing cytosolic DNA or the presence of various intracellular pathogens, STING mediates IFN-β production to mount an immune response. Importantly, it has been demonstrated that efficient tumor-specific CD8 T cell responses require STING-dependent IFN-β production in the TME, and STING activation by DC. Therefore, the use of a STING agonists to activate this pathway could function as a potential therapy for various cancers [156]. Several preclinical studies are evaluating the activity of various STING agonists in combination with immune checkpoint blockade or PARPi as therapies for EOC. A recent in vivo analysis of STING agonist along with carboplatin chemotherapy and PD-1 ICB was performed using the ID8-*Trp53^−/−^* immunocompetent murine model of high-grade serous ovarian cancer. Evidence from this preclinical study showed that treatment with STING agonist resulted in decreased ascites accumulation and decreased tumor burden in mice. Notably, survival was the longest for mice treated with combination STING agonist, carboplatin and anti-PD-1 antibody [157]. These data are in line with results showing STING engagement following chemotherapy and provide the rationale for the future development of STING agonists for HGSOC [23]. Clinical translation of results obtained from animal models has been met with mixed success, partly because of poor stability properties of current STING agonists, requiring intratumoral, rather than systemic administration. The current focus of STING agonist development is on agonists formulated for systemic administration and a small number of intravenous STING agonists are currently being evaluated in clinical trials. Two non-nucleotide, small-molecule STING agonist, termed MSA-2 and SR-717, which produced substantial efficacy when administered systemically, were recently reported by Pan et al. and Chin et al., respectively [158,159]. These compounds stabilize STING in its closed conformation, leading to downstream signaling events that trigger increased expression of IFN-β and interleukin-6 (IL-6). The presence of these proinflammatory cytokines promotes DC maturation and facilitates DC priming of tumor antigen-specific CD8+ T cells in the tumor-draining lymph node. These encouraging results pave the way for future development of non-nucleotide small-molecule STING agonists that can be administered systemically in many solid tumor types, including EOC.

## 9. Conclusions

Immune-based strategies are an exciting and promising approach for disease management in EOC. Single agent immune approaches trigger modest effects, although their efficacy can be improved by combinations with drugs or immune biologics that act synergistically. We discussed here some of the most advanced bench-to-bedside developments that test ICB, vaccines or cellular therapies, all with high potential to positively impact clinical outcome. One of the major barriers to immune therapy is the very suppressive immune microenvironment in OC, which translates into the inability of the new, therapy-induced anti-tumor effectors to travel to the tumor site and infiltrate the TME. To clear this barrier, further explorations of the dynamic interaction between immune cell subsets, and between immune cell -tumor cell and immune cell-stroma cell interactions are needed. In addition to the widely used “omics” platforms, advanced multiplex tissue profiling methods that reveal detailed spatial information of the TME hold promise and may spearhead efforts on biomarker discovery and patient stratification [160].

Going forward, several challenges remain, including but not limited to the standardization of patient stratification using predictive biomarkers of response (such as immunoscore, BRCA and DNA HR status), mitigation of sometimes significant, treatment-induced side effects, and development of new, safe and effective combination therapies.

## Figures and Tables

**Table 1:** Summary of phase III EOC clinical trials testing tumor-targeting monoclonal antibodies (mAb).

Agent (Target)	Study Phase	Combination	Outcome	Reference	NCT
Oregovomab (CA125)	III	No	No significant difference between groups for time to relapse (TTR). Median TTR of 10.3 months [95% CI, 9.7 to 13.0] for oregovomab and 12.9 months [95% CI, 10.1 to 17.4] for placebo (*p* = 0.29, log-rank test)	[31]	NCT00050375
Farletuzumab (FRa)	III	Carboplatin and paclitaxel	No significant difference in progression free survival (PFS). HR 0.99 [95% CI, 0.81 to 1.21] and 0.86 [95% CI, 0.70 to 1.06] for farletuzumab 1.25 mg/kg (*p* = 0.9025, two-sided log-rank test) and 2.5 mg/kg (*p* = 0.1521) vs. placebo, respectively.	[32]	NCT00849667
Mirvetuximab soravtansine IMGN853 (FRa)	III	No	ORR of 22% (12% for chemo alone) In the high FRa expression group ORR was 24% (10% for chemo alone)	[33]	NCT02631876
Catumaxomab (EpCAM)	II/III	Paracentesis	Significant puncture-free survival benefit for catumaxomab + paracentesis vs. paracentesis alone in OC (52 vs. 11 days, respectively, *p* < 0.0001). No significant survival benefit for catumaxomab + paracentesis vs. paracentesis alone in OC (110 vs. 81 days, respectively, *p* = 0.1543)	[34]	NCT00836654
Bevacizumab (VEGF)	III III	Carboplatin and paclitaxel Olaparib (PARPi)	No survival benefit for bevacizumab vs. chemotherapy. Median OS for stage IV disease: bevacizumab + chemo 42.8 months (32.6 for chemo alone, HR, 0.75; 95% CI, 0.59 to 0.95). Median PFS was 22.1 vs. 16.6 months for olaparib vs. placebo (HR, 0.59; 95% CI, 0.49 to 0.72; *p* < 0.001). Median PFS for BRCA mutated HRD-positive tumors was 37.2 vs. 21.7 months for olaparib vs. placebo (HR, 0.33; 95% CI, 0.25 to 0.45). Median PFS for non-BRCA HRD-positive tumors was 28.1 vs. 16.6 months for olaparib vs. placebo (HR, 0.43; 95% CI, 0.28 to 0.66).	[35,36]	NCT00262847 NCT02477644

**Table 2:** Summary of results from trials testing immune checkpoint blockade in EOC.

AGENT (Target)	Study Phase	Combo	Cohorts	Outcome	PD-L1	Ref	NCT
Ipilimumab (CTLA-4)	II	None	38/40 patients did not complete the trial due to toxicity, progression or death	N/A		[59]	NCT01611558
Nivolumab (PD-1)	II	None	A—Low dose—1 mg/kg B—High dose—3 mg/kg 2 patients with CR	A—ORR 10%, DCR 50%, medium PFS 3.5 months, median OS 16.1 months B—ORR 20%, DCR 40%, medium PFS 3.0 months	High expression—16/20, scores +2 to +3 Low expression—4/20, score of +1	[60]	UMIN000005714
Pembrolizumab (PD-1)	II	None	A—received </=2 prior chemotherapy lines for recurrent AOC B—3–5 prior chemotherapy lines	A—ORR 8.1%, median OS 18.7 mo B—ORR 9.9%, median OS 17.6 mo Patients with higher PD-L1 expression had better response in both cohorts		[61]	NCT02674061
Avelumab (PD-L1)	III III	Carboplatin Doxorubicin	A—Carboplatin B—Carboplatin + avelumab 10mg/kg C—Carboplatin + avelumab, w/maintenance A—avelumab 10 mg/kg B—avelumab 10 mg/kg + PLD 40 mg/m^2^ C—PLD 40 mg/m^2^	A—PFS N/A, ORR 27.8% B—PFS 16.8 mo, ORR 25.9% C—PFS 18.1 mo ORR 31.1% A—median PFS 1.9 mo, median OS 11.8, ORR 3.7% B—median PFS 3.7 mo, median OS 15.7 mo, ORR 13.3% C—median PFS 3.5, median OS 13.1 mo, ORR 4.2% ORR 18.5% for PD-L1 pos tumors, 3.4% PD-L1 neg tumors	PD-L1 positive if expression >/=1% of tumor cells or >/=5% immune cells PD-L1 positive if expression >/=1% of tumor cells or >/=5% immune cells	[62,63]	NCT02718417 NCT02580058
